# Effect of Different Carbon Sources on Biosurfactants' Production by Three Strains of* Lactobacillus* spp.

**DOI:** 10.1155/2018/5034783

**Published:** 2018-02-13

**Authors:** Tene Hippolyte Mouafo, Augustin Mbawala, Robert Ndjouenkeu

**Affiliations:** ^1^Centre for Research on Food and Nutrition, Institute of Medical Research and Medicinal Plants Studies, P.O. Box 6163, Yaounde, Cameroon; ^2^Department of Food Sciences and Nutrition, National School of Agro-Industrial Sciences, University of Ngaoundere, P.O. Box 455, Ngaoundere, Cameroon

## Abstract

The potential of three indigenous bacterial strains (*Lactobacillus delbrueckii* N2,* Lactobacillus cellobiosus* TM1, and* Lactobacillus plantarum* G88) for the production of biosurfactants using sugar cane molasses or glycerol as substrates was investigated through emulsifying, surface tension, and antimicrobial activities. The different biosurfactants produced with molasses as substrate exhibited high surface tension reduction from 72 mN/m to values ranged from 47.50 ± 1.78 to 41.90 ± 0.79 mN/m and high emulsification index ranging from 49.89 ± 5.28 to 81.00 ± 1.14%. Whatever the* Lactobacillus* strain or the substrate used, the biosurfactants produced showed antimicrobial activities against* Candida albicans* LV1, some pathogenic and/or spoilage Gram-positive and Gram-negative bacteria. The yields of biosurfactants with molasses (2.43 ± 0.09 to 3.03 ± 0.09 g/L) or glycerol (2.32 ± 0.19 to 2.82 ± 0.05 g/L) were significantly (*p* < 0.05) high compared to those obtained with MRS broth as substrate (0.30 ± 0.02 to 0.51 ± 0.09 g/L). Preliminary characterization of crude biosurfactants reveals that they are mainly glycoproteins and glycolipids with molasses and glycerol as substrate, respectively. Therefore, sugar cane molasses or glycerol can effectively be used by* Lactobacillus* strains as low-cost substrates to increase their biosurfactants production.

## 1. Introduction

Surfactants are amphiphilic molecules that, due to their structure, tend to accumulate at the interfaces between fluid phases with different polarities (e.g., oil-water or air-water) and thereby reduce surface and interfacial tensions [1]. They are very important chemical compounds which are used in a variety of products with very high volume because of their domestic and industrial applications [2, 3]. Nowadays, with current advances in biotechnology and due to the increasing environmental awareness, attention has shifted to the alternative environmental friendly process for production of different types of biosurfactants from bio-based resources as microorganisms [4, 5]. Biosurfactants are therefore the natural choice for such processes as they possess a lot of advantages over synthetic surfactants, such as lower toxicity, biodegradability, and effectiveness at a wide range of pH and temperature values [6, 7]. Bacterial biosurfactants were initially proposed to function as emulsifiers of biodegradable hydrocarbons [8]. However, a wide variety of roles for biosurfactants have been described including their antimicrobial [9–12], antiadhesive [13, 14], emulsifying [5, 12], and antioxidant properties [15]. Furthermore, a renewed interest in their discovery has been sparked [16, 17], especially to those produced by lactic acid bacteria due to their GRAS (generally recognized as safe) statute and their well-known probiotic effect [18, 19]. The most known lactobacilli producing biosurfactants were isolated from the urogenital and gastrointestinal tract microbiota of humans [20–22]. They have been reported to inhibit pathogenic bacteria and fungi and to reduce adhesion of pathogenic microorganisms to glass, silicone rubber, and surgical implants [13, 20, 21, 23]. The characterization of biosurfactants produced by lactobacilli reveals that they are generally glycolipid, lipopeptide, glycoprotein, glycolipopeptide, and phosphoglycoprotein independent of the strain, the culture conditions, and the medium composition [20, 21, 24, 24, 25]. The emulsifying, antimicrobial, and antiadhesive activities of lactobacilli biosurfactants' are also well documented [11, 14, 18, 26, 27].

Despite the numerous advantages of lactobacilli biosurfactants, they are less effective in reducing surface tension of water (approximately 36–40 mN/m) compared to other biosurfactants which are able to reach values lower than 30 mN/m. Moreover, they are not yet used intensively for industrial productions, since expensive substrates are required for their production and they present relatively low productivities (20–100 g/L) [13, 20, 27], which hampers their widespread use and commercialization [5, 28, 29]. As the culture medium can account for up to 30–50% of the overall production cost of biosurfactants, the replacement of expensive synthetic media by cheaper agroindustrial wastes and by-products can contribute to the reduction in production cost and increase their competitiveness [5, 29, 30]. This leads to research for alternative and less costly substrates which could be used as substitutes [28].

Studies carried out by Mbawala and Mouafo [31] and Mbawala et al. [11] enabled the isolation from acidic curdled milk* (pendidam)* of three strains of* Lactobacillus *spp. which produced biosurfactants while using whey as substrate with yield twice as high as those obtained with MRS broth. This yield still remains relatively low from an economic point of view, if the local availability of the whey used is considered. However, the possibility of producing biosurfactants from other local biological substrates leads to the search of less costly and more readily available substrates in Cameroon; this also will lead to increase in the yield of production. In this light, several studies have been carried out using sugar cane molasses as substrate to increase the production yields of biosurfactants [32, 33]. Henkel et al. [30] and Khopade et al. [34] have shown glycerol as a promising substrate for biosurfactants production. Glycerol represents a by-product which the amount of wastes has been raised year by year through the increasing production of biodiesel and other oleochemicals. Sugar cane processing industries which produce large amount of molasses are available in Cameroon. It therefore appears to be very interesting to carry out a test on these substrates while using* Lactobacillus *spp. strains in the production process. The present work aims to evaluate the production of biosurfactants by three strains of* Lactobacillus* spp. while using sugar cane molasses or glycerol as substrates.

## 2. Materials and Methods

### 2.1. Bacterial Strains

The biosurfactant-producing strains* Lactobacillus cellobiosus* TM1,* Lactobacillus delbrueckii* N2, and* Lactobacillus plantarum* G88 were isolated and identified in previous works [16, 25]. The bacteria were kept frozen in conventional synthetic Man, Rogosa, and Sharpe (MRS) broth (LiofilChem, Italy) with the addition of glycerol 20% (v/v). Before each experiment, the bacteria were cultivated twice on MRS broth at 37°C for 18 h.

The following microbial strains previously isolated from ground beef sold in Adamawa region of Cameroon and identified (Unpublished data) were used for antimicrobial assays. They included Gram-negative microorganisms like* Escherichia coli* E2B,* Escherichia coli* E2R,* Escherichia coli* E5,* Escherichia coli* E6,* Pseudomonas aeruginosa* PSB2,* Pseudomonas aeruginosa* PSB1,* Pseudomonas aeruginosa* PSR1,* Pseudomonas aeruginosa* PSR2,* Pseudomonas putida* PS3,* Pseudomonas putida* PSJ1,* Pseudomonas putida* PSV1,* Pseudomonas putida* PSV2,* Salmonella* S5,* Salmonella* SL2, Gram-positive microorganisms like* Bacillus* sp. BC1 and* Staphylococcus aureus* STP1, and yeasts like* Candida albicans* LV1.

### 2.2. Sugar Cane Molasses

Sugar cane molasses was provided by SOSUCAM, the Cameroon Sugar Company of Mbanjock (Central region of Cameroon), and it was evaluated as a substrate for biosurfactants production by* Lactobacillus* strains. Before use, it was clarified according to the method described by Tazdait et al. [35]. The clarification was done chemically by adding 3 mL of concentrated H_2_SO_4_ to 1 Kg of molasses mixed with 1000 mL of distilled water, to reach pH 3.5. Then, the mixture was heated in a water bath to boil for 30 minutes, and after being cooled, it was completed to 2000 mL and kept overnight at 4°C. Thereafter, it was centrifuged (6000 g, 4°C, 10 min; Biofuge R apparatus) and the supernatant was treated with NaOH 1 N, to reach pH 10 and centrifuged (6000 g, 4°C, 10 min). The obtained supernatant was stored at room temperature. The clarified molasses was characterized (pH, Brix, dry matter, total and reducing sugar contents, total protein, and ash content).

### 2.3. Glycerol

Glycerol was obtained from the Food Microbiology and Biotechnology Lab of the National School of Agro-Industrial Sciences, University of Ngaoundere (Cameroon).

### 2.4. Production of Biosurfactants

The method described by Rodrigues et al. [32] with some modifications was used. For crude biosurfactants production by the three* Lactobacillus' *strains, the culture media were prepared as follows: for 1 L of broth, K_2_HPO_4_: 1.6 g/L, KH_2_PO_4_: 0.4 g/L, NaCl: 0.1 g/L, MgSO_4_7H_2_O: 0.1 g/L, CaCl_2_: 0.02 g/L, 1 mL of trace element (solution containing in mg per 100 mL CuSO_4_5H_2_O: 0.5; H_3_BO_3_: 1.0; MnSO_4_5H_2_O: 1.0; ZnSO_4_: 0.7; and MoO_3_: 1.0), peptone: 8 g/L, yeast extract: 5 g/L, and substrate (sugar cane molasses or glycerol) 9% (w/v). The pH of the broth was adjusted to 6.7 and MRS broth was used as standard culture medium.

600 mL of prepared and sterilized broth was inoculated with 15 mL of an overnight subculture of* Lactobacillus* spp. strains (10^6^ CFU/mL) and incubated for 72 h at 37°C under agitation (150 oscillations/min) in a shaker incubator (Kottermann® D-3162, Germany). After incubation, the cultures were centrifuged (20 min, 4°C, 65000 g; Biofuge R Apparatus), filtered (0.22 *μ*m; Millipore, Sartorius Stedim, Germany), treated with catalase (catalase 2.000 U/mg in phosphate buffer 10 mM, pH 7) and proteinase K (50 UI/mL in phosphate buffer 50 mM, pH 6,5), neutralized to pH 7 with NaOH 0.1 N, and stored for the detection of biosurfactants.

### 2.5. Screening of Biosurfactants Produced

To evaluate the production of biosurfactants by the different* Lactobacillus* strains while using sugar cane molasses and glycerol as substrates, emulsification activity, surface activities, and antimicrobial activity of the stored supernatants were determined.

#### 2.5.1. Emulsification Activity (E24)

The emulsifying capacity was evaluated by an emulsification index (E24). The E24 was determined by adding 1 mL of refined palm oil and 1 mL of the bacterial supernatant in a test tube, vortexed (vibrant agitator, FIRLABO.sa) for 2 min, and allowed standing for 24 h. Distilled water and SDS 1% (w/v) were used as negative and positive control, respectively. The emulsion activity was investigated after 24 h and the percentage of emulsification index was calculated by using the following equation [36]:

(1) $$\begin{array}{c} E24=\left(\;\frac{Height\;\;of\;\;emulsion\;\left(cm\right)}{Height\;\;of\;\;the\;\;total\;\;mixture\;\left(cm\right)}\;\right)\times 100. \end{array}$$

#### 2.5.2. Surface Activities

To determine the surface activities of the obtained supernatants, drop collapse test and surface tension measurements were carried out.

*(1) Drop Collapse Test*. This assay relies on the destabilization of liquid droplets by biosurfactants. Drops of a cell suspension or of culture supernatant are placed on an oil-coated, solid surface. If the liquid does not contain biosurfactants, the polar water molecules are repelled from the hydrophobic surface and the drops remain stable. If the liquid contains biosurfactants, the drops spread or even collapse because the force or interfacial tension between the liquid drop and the hydrophobic surface is reduced. The stability of drops depends on biosurfactants concentration and correlates with surface and interfacial tension. To this end, 2 *μ*L of refined palm oil was used to coat the surface of a glass plate and left to equilibrate for 24 h. 20 *μ*L of supernatants was added to the surface of the coated plate and drop size was observed after 1 min with the aid of a magnifying glass. The result was considered positive for biosurfactants production when the drop was flat and the cultures that gave rounded drops were scored as negative, indicative of the lack of biosurfactant production [37]. Then, the plates were dried and the drop diameter was recorded. Distilled water and SDS 1% (w/v) were used as negative and positive control, respectively.

*(2) Surface Tension Measurement*. The “Du-Nouy-Ring method” as described by Abouseoud et al. [36] and Devesa-Rey et al. [38] was used to determine the surface tension of the different supernatants. The surface tension (ST) was measured by means of a tensiometer (3B Scientific® product U20030) with the ring method, using a platinum ring (De Noüy) 3 cm in diameter at a temperature of 25°C. The ring was placed just below the surface of the supernatant solutions; subsequently, the force to move this ring from the liquid phase to the air phase was measured and used to calculate the surface tension as follows:

(2) $$\begin{array}{c} \text{ST}=\left(\;\frac{F-P_{0}}{4\pi r}\;\right)\times 1000, \end{array}$$

where *F* represents the force measured, *P*_0_ the force read before removing the ring, and *r* the radius of the ring.

The presence of biosurfactants in the solution was confirmed based on a decrease in the value of surface tension of the supernatants against the control (distilled water). All measurements were repeated three times and their mean values were taken.

#### 2.5.3. Antimicrobial Activity

To assess the antimicrobial activity of the supernatants, the well diffusion method described by Topisirovic et al. [39] was used. All the tested strains were cultured in Trypticase soy broth (LiofilChem, Italy) at 37°C for 24 h. Then, the different broths were streaked on nutrient agar and incubated and a colony of each strain was introduced in a tube containing 15 mL of Trypticase soy broth. After incubation at 37°C for 18 h, the microbial load of each tube was determined.

Sterile Mueller-Hinton Agar was poured into Petri dishes and allowed to cool at room temperature. Then, 0.1 mL of the different bacterial suspensions (10^6^ CFU/mL) was spread at the surface of the media and the Petri dishes were left on the working surface for one hour in order to allow the suspension to dry. Thereafter, well of 6 mm in diameter was digged and 25 *μ*L of the different supernatants was introduced. The Petri dishes were stored at 4°C for 4 h before being incubated at the respective optimal growth temperature of each microorganism for 24 h. After incubation, the diameter of the inhibition zone was measured.

### 2.6. Extraction of Biosurfactants

After detecting the production of biosurfactants by the* Lactobacillus* spp. strains using sugar cane molasses and glycerol as substrates, the produced compounds were extracted from the supernatants using the solvent method described by Fracchia et al. [23]. The supernatants were acidified to pH 2 with 6 N HCl, stored overnight at 4°C, and extracted three times with equal volume of ethyl acetate/methanol (4 : 1). The organic fraction was evaporated to dryness under vacuum condition in a rotavapor (Heidolph VV60), and the crude biosurfactants was collected, weighed, and stored at room temperature.

### 2.7. Preliminary Characterization of Crude Biosurfactants

#### 2.7.1. Chemical Composition

The chemical composition of crude biosurfactants produced by lactobacilli strains using sugar cane molasse or glycerol as substrates was assessed following standard methods. The total proteins content was determined according to the Kjeldahl method [40]. The total sugars content was evaluated by the phenol-sulphuric method described by Dubois et al. [41] using glucose as the standard. Lipid content was estimated adopting the procedure of Bourely [42].

#### 2.7.2. Presence of Phosphates

The presence of phosphates in the composition of biosurfactants was assessed by the method of Okpokwasili and Ibiene [43]. Six drops of nitric acid 6 M were added to 2 mL of biosurfactants' solutions 1% (w/v) and heated for 30 min at 70°C. Then, a solution of ammonium molybdate 5% (w/v) was added drop by drop until a yellow colour appears followed by the formation of a yellow precipitate which indicated the presence of phospholipids in the biosurfactants' solutions. Lecithin solution (1%, w/v) was used as control.

#### 2.7.3. Emulsifying Activities

The method of Abouseoud et al. [36] described above was used to determine emulsification index of crude biosurfactants (1%, w/v). To assess emulsion's stability, emulsification index was measured after 1, 24, 48, 72, and 96 hours of storage at room temperature (24 ± 2°C). SDS 1% (w/v) and distilled water were used as positive and negative controls, respectively.

#### 2.7.4. Surface Tension and Critical Micelle Concentration

Critical micelle concentration (CMC) is known as the concentration of an amphiphilic component in solution at which the formation of micelles is spontaneously initiated. It is important for several biosurfactants applications to establish their CMC, as above this concentration no further effect is expected in the surface activity. The CMC was determined by plotting the surface tension as a function of biosurfactants concentration and was found at the point corresponding to the lower concentration in biosurfactants for which no significant variation in surface tension was observed. Biosurfactants were serially diluted in phosphate-buffered saline (PBS) at different concentrations (from 0.2 to 40 mg/mL), and the surface tension of each sample was measured as described above.

### 2.8. Statistical Analysis

All measurements were done in triplicate and data presented are mean values ± standard deviation. A one-way analysis of variance (ANOVA) was used to study the significant difference between mean values, at a significance level of *α* = 0.05. Mean comparisons were carried out using Duncan's test at a level of significance *p* < 0.05. Principal component analysis was carried out to visualize the correlation between the different parameters assessed for biosurfactants detection.

## 3. Results and Discussion

### 3.1. Physicochemical Analysis of Sugar Cane Molasses

The results of the physicochemical analysis of sugar cane molasses are presented in Table 1. From Table 1, sugar cane molasses of SOSUCAM Mbanjock have a pH value of 5.6 and a Brix of 79.24. The sugar cane molasses is composed of total sugars of 51.23 g/100 g dry matter (DM), total proteins of 2.59 g/100 g DM, and an ash content of 3.56 g/100 g DM. These results are included to the range of values reported by Makkar and Cameotra [44] who found that sugar cane molasses generally consists of 48–56 g/100 g DM of total sugar and 2–4 g/100 g DM of proteins. In the same way, Banat et al. [45] have found a total sugar content of 48–56 g/100 g DM and a total protein content of 2.5 g/100 g DM in sugar cane molasses. The results of the characterization of sugar cane molasses obtained in this study suggest the suitability of this substrate for fermentative processes involving lactobacilli.

### 3.2. Screening of Biosurfactants

#### 3.2.1. Emulsification Activity

The emulsification index of the supernatants obtained with glycerol or sugar cane molasses as substrates is presented in Table 2. All the supernatants show an emulsification activity with emulsification index ranging from 41.81 ± 2.56 to 81.00 ± 1.14%. The emulsification index varies significantly (*p* < 0.05) with the biosurfactants producing strains and the substrates used. Sugar cane molasses had the highest emulsification index, suggesting its good ability to be used as substrate in the production of biosurfactants. This high emulsification index could be due to the composition of sugar cane molasses which contains besides sugar other nutrients (proteins, mineral) which can stimulate the production of biosurfactants. Das et al. [46] also reported that emulsification index is proportional to the biosurfactants concentration. The emulsification activity of lactic acid bacteria biosurfactants as reported by Brzozowski et al. [47] showed that* Lb. rhamnosus* and* Lb. fermentum *produced biosurfactants had good emulsifying ability. Kermanshahi and Peymanfar [48] found that the culture supernatants of* Lactobacillus* spp. had an emulsification index ranging from 64 to 72%. Salman and Alimar [49] have also found that the supernatants of* Lactobacillus rhamnosus* had an emulsification index of 50%.

The emulsification index obtained with SDS was significantly higher than those obtained with the supernatants whatever the biosurfactants producing strains or the substrates used. This difference may be due to the amount of biosurfactants in the supernatants compared to SDS where a concentration of 10 g/L was used.

#### 3.2.2. Surface Activities of Biosurfactants

*(1) Drop Collapse Test*. The drop collapse test was conducted for the primary screening of biosurfactants production. This qualitative test is indicative of the surface and wetting activities [37] and it represents an indirect measurement of surface activity of a biosurfactant. In the present study, surface activities of the supernatants were investigated in comparison with that of SDS. From Table 3 it is clear that all the supernatants were positive to this test with drop diameters ranging from 12.80 ± 0.98 to 19.00 ± 1.41 mm. The biosurfactants activities of the supernatants represented by drop diameters showed that supernatants used really contained biosurfactants since the force or interfacial tension between the drop containing the biosurfactants and the oiled surface was reduced and resulted in the spread of the drop. Rodrigues et al. [13, 27] and Walencka et al. [50] also demonstrated that surface tension was reduced by biosurfactants of lactic acid bacteria.

The drop diameters obtained for all the supernatants were significantly (*p* < 0.05) higher than those obtained with distilled water. Youssef et al. [37] and Plaza et al. [51] pointed out that a test is considered positive if the drop diameter of the culture is greater of 0.5 mm than the diameter of the distilled water drop. In accordance with their studies, sugar cane molasses and glycerol can be considered as good substrates to improve the production of the biosurfactants by* Lactobacillus* strains. The amount of surface-active compounds present in each supernatant could explain the significant difference (*p* < 0.05) observed between the supernatants drop diameters and the SDS drop diameter where concentration was 10 g/L.

*(2) Surface Tension Measurement*. In order to complete the surface activity observed with drop collapse test, surface tension of the different supernatants was measured and the results are presented in Table 4. The results showed that biosurfactants present in the different supernatants caused a significant (*p* < 0.05) reduction of surface tensions. A decrease in surface tension of molasses made broth from 59.71 mN/m to values ranging from 47.50 mN/m to 41.90 mN/m was observed. Concerning broth made with glycerol as substrate, a decrease from 60.17 mN/m to values ranging from 46.20 mN/m to 49.00 mN/m was noticed. A lower reduction of surface tension was observed with* Lb. plantarum* G88 while using glycerol as substrate and the higher reduction was observed with* Lb. delbrueckii* N2 while using molasses as substrate. The high surface activity of the different supernatants observed in the present study could be explained by the fact that supernatants may probably contain biosurfactants composed of a mixture of several compounds with surface activity as reported by Brzozowski et al. [47] in their study with* Lb. rhamnosus.*

The results obtained in this study are in accordance with the data previously reported for other lactobacilli. Rodrıguez et al. [52] obtained a reduction of surface tension to 41.1 mN/m with biosurfactants of* Lactococcus lactis*. Madhu and Prapulla [24] obtained a reduction of surface tension to 44.3 mN/m with biosurfactants of* L. plantarum* CFR2194. Sharma et al. [19] obtained a reduction of surface tension to 39.5 mN/m with biosurfactants of* L. helveticus* MRTL 91. However, Busscher et al. [53] established a minimum decrease in surface tension as 8 mN/m to distinguish between biosurfactants producing and nonproducing organisms. Taking into account this value, all the lactobacilli strains used in the present study could be considered as an excreted biosurfactant producer and molasses or glycerol as good substrates for the production of biosurfactants by these strains.

When the substrates are considered, sugar cane molasses seems to be the best among the studied substrates. It enables the highest reduction of surface tension and the highest emulsification index.* Lb*.* delbrueckii* N2 seems to be the most producing strain. In order to confirm this hypothesis, biosurfactants were extracted from each supernatant.

#### 3.2.3. Antimicrobial Activity

Table 5 presents the inhibition diameters of the different supernatants against some pathogenic and spoilage microorganisms isolated from fresh ground beef sold in Adamawa region of Cameroon. All the supernatants were actives against the test germs with inhibition diameter which varies significantly (*p* < 0.05) from one test germ to another and from one substrate to another. These antimicrobial activities could be explained by the presence of biosurfactants in the supernatants which have initiated their interaction with the cytoplasmic membrane by binding to the phospholipid surface through electrostatic forces. They are then absorbed in the hydrophobic core of the membrane perturbing the packing of the lipids, leading to the dissolution of the proton motive force and leakage of essential molecules [54, 55]. The antimicrobial activities observed could also be an outcome of the adhesion property of these surface-active agents to the cell surfaces instigating decline of cell membrane integrity and leading to subsequent collapse of the nutrition cycle [56].

The antimicrobial activity of lactobacilli biosurfactants against Gram-positive and Gram-negative bacteria have also been reported in the literature. Gudiña et al. [14] reported that biosurfactants produced by* Lactobacillus paracasei *are actives against* E. coli, P. aeruginosa, S. aureus, Staphylococcus epidermidis, Streptococcus agalactiae*, and* Streptococcus pyogenes.* Sharma and Saharan [57] also showed that culture supernatants of* Lactobacillus* strains are active against* E. coli*,* P. aeruginosa*,* Staphylococcus* spp., and* Salmonella typhi* with inhibition diameter ranging from 2 to 21 mm. The activities were attributed to biosurfactants molecules present in the culture supernatants. From Table 5, whatever the substrate used, the yeast strain* Candida albicans* LV1 was more resistant to the antimicrobial activity of all supernatants. The fact that yeast strains were more resistant than the other test germs could be due to the higher amount of anionic phospholipids in prokaryotic membranes, compounds which facilitate their interaction with biosurfactants molecules [58, 59].

Gram-positive bacteria tested (*Bacillus* sp. BC1 and* Staphylococcus aureus* STP1) were more sensitive to the activity of biosurfactants produced with glycerol used as substrate. The higher sensitivity of Gram-positive bacteria observed in this study could be explained by the fact that Gram-negative bacteria tend to resist against many antimicrobial substances due to the lipopolysaccharides layer present in their external membrane and which act as an effective permeability barrier against hydrophobic molecules and macromolecules [60].

### 3.3. Biosurfactants Production

Table 6 shows that the yields of biosurfactants extracted from the different supernatants vary significantly (*p* < 0.05) from one biosurfactants producing strain to another and from one substrate to another. The yields of biosurfactants with molasses or glycerol substrates were higher than that of MRS broth, suggesting therefore their good ability to be used as substrate for a low-cost production of biosurfactants by lactobacilli strains. The highest yield of biosurfactants was recorded with molasses as substrate. It could be explained by the presence of compounds other than sugar in the molasses which may have contributed to the increased production of biosurfactants. The yield obtained in this study was high than those reported in the literature with lactobacilli. Sharma et al. [19] found a yield of 0.80 g/L with* Lactobacillus helveticus* MRTL91 while using cheese whey as an alternative nutrient source. Distilled grape marc residues were used by* Lactobacillus pentosus* to produce 4.8 mg/L of biosurfactants [61]. Thavasi et al. [29] in their studies established the production of 5.35 mg/L of biosurfactants by* Lactobacillus delbrueckii* while using peanut oil cake as a low-cost substrate.

Considering the biosurfactants producing strains,* Lb. delbrueckii* N2 produced the highest yield, observed when molasses or MRS broth is used as substrates. However, when glycerol is used as substrate* Lb*.* cellobiosus* TM1 was the strain with the higher yield of biosurfactants. This variability in yield with the strains and substrates could be explained by the fact that the metabolism of substrates to synthetize biosurfactants depends on the intrinsic enzymatic package of each strain [62].

The yields of biosurfactants obtained in this study with lactobacilli strains when sugar cane molasses is used as substrate are high compared to those obtained with other authors in literature. Rodrigues et al. [32] reported in their study with* Streptococcus thermophilus* A a yield of 1.401 g/L and with* Lactococcus lactis* 53 a yield of 1.735 g/L. This difference could be explained by the metabolic activity of the strains used which varied from a strain to another [62].

In previous study carried out by Mbawala et al. [11] with* Lb*.* cellobiosus* TM1 and* Lb*.* delbrueckii* N2 while using* pendidam* whey as substrate, a biosurfactants yield of 1.20 and 1.1 g/L was obtained, respectively. These yields are very low compared to those obtained in this study with the same strains while using molasses or glycerol as substrate. This significant difference (*p* < 0.05) could be explained by the fact that, in the present work, broth was supplemented with yeast extract and peptone which according to Gudiña et al. [63] was essential component for bacterial growth and most important factors for biosurfactants production by lactobacilli strains, respectively.

The yields of biosurfactants obtained in this study with lactobacilli strains while using MRS broth as substrate (0.30 to 0.51 g/L) were in accordance with those obtained by Rodrigues et al. [32] with* Lactococcus lactis* and* Streptococcus thermophilus *A (0.48 g/L) and by Fracchia et al. [23] with* Lactobacillus *sp. CV8LAC (0.48 g/L) when MRS broth was used as substrate.

### 3.4. Correlation Analysis

**Figure 1:**
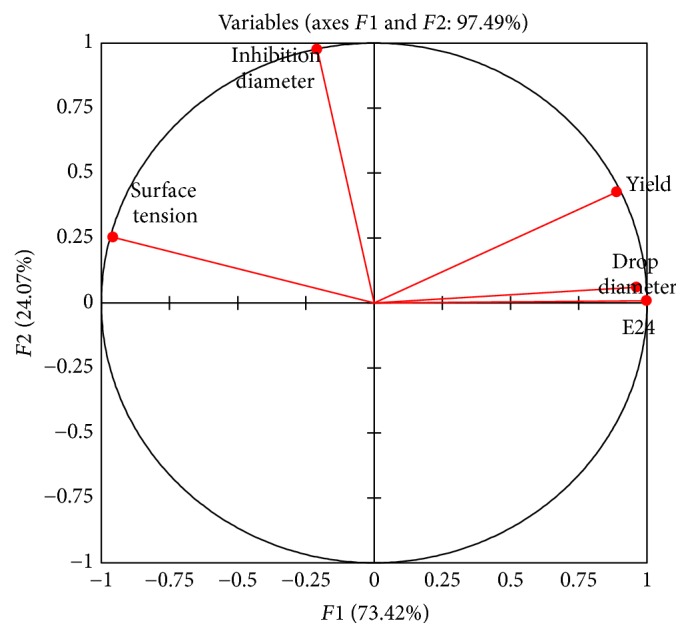
Correlation circle of the different parameters assessed to screen the production of biosurfactants.

Principal component analysis was carried out to visualize the way that the different parameters assessed to screen the production of biosurfactants by lactobacilli strains are correlated. The obtained correlation circle is shown in Figure 1.

It comes from this analysis that all variables contribute to 97.49% of the variations observed. *F*1 axis contributes to 73.42% and *F*2 axis contributes to 24.07%. Three groups can easily be distinguished. The first group which is made up with yield, drop diameter, and E24 shows that the yield of biosurfactants is positively correlated to the drop diameter (*r* = 0.839) and the emulsification index (*r* = 0.895). The second group with surface tension is opposite to the first group. These results can be explained by the fact that the more the surface tension of the supernatants is reduced (low), the more its yield in the supernatant is important (high). A negative correlation between surface tension measurement and yields of biosurfactants was also reported in the literature by many authors [11, 13, 50]. The third group represented by the inhibition diameter was slightly correlated (*r* = 0.225) with yield of biosurfactants. As it is known that the antimicrobial activity is proportional (positively correlated) to the quantity of biosurfactants, the results obtained in the present study could be explained by the fact that, besides the quantity of biosurfactants, its nature, composition, and structure could also play a role in the antimicrobial mechanism. Correlation between the different variables used to screen the production of biosurfactants is presented in Table 7.

**Figure 2:**
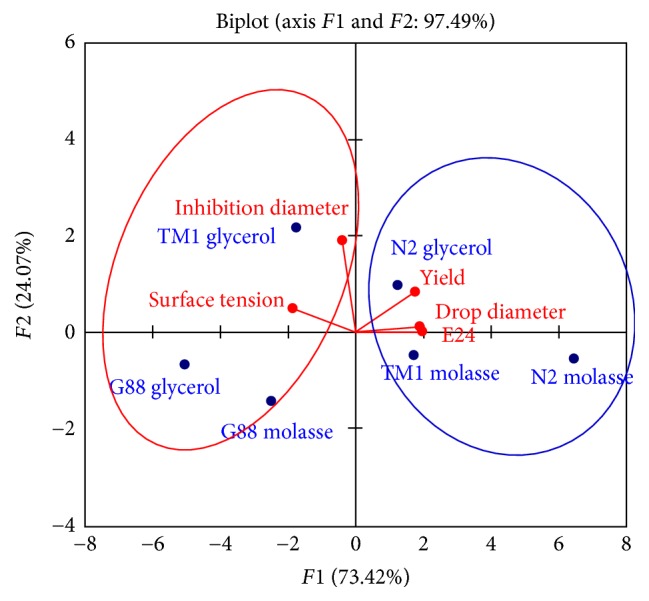
Principal component analysis of the lactobacilli strains, the substrate, and biosurfactants parameters.

The projection of all variables in the axis system (Figure 2) shows an association between the lactobacilli strains and their ability to use substrates for the production of biosurfactants with specific properties. Two main groups can be observed: the first shows that the use of sugar cane molasses as substrate by the strains* Lb*.* cellobiosus* TM1 and* Lb*.* delbrueckii* N2 is positively associated with yield, drops diameter, and emulsification index of biosurfactants. However, the second group shows that utilization of glycerol as substrate by* Lb*.* cellobiosus* TM1 is positively associated with the production of biosurfactants with high antimicrobial activity. The second group also shows that the use of glycerol or sugar cane molasses by* Lb. plantarum* G88 is negatively associated with antimicrobial activity and surface tension as well as yield and emulsification index. This result means that* Lb. plantarum* G88 whatever the carbon source used produces a low level of biosurfactants.

### 3.5. Preliminary Characterization of Crude Biosurfactants

#### 3.5.1. Chemical Composition

Table 8 presents the chemical composition of the crude biosurfactants produced by lactobacilli strains while using sugar cane molasses or glycerol as substrate.

The high protein contents (52.93 ± 1.27 and 63.64 ± 0.24 g/100 gMS) were obtained with biosurfactants produced by* Lb. cellobiosus* TM1 and* Lb. delbrueckii *N2 while using sugar cane molasses as substrate, respectively. Sugars were present in all crude biosurfactants analyzed at concentrations ranging from 27.10 ± 0.36 to 51.13 ± 0.92 g/100 gMS. The presence of sugar in all biosurfactants produced independent of the strain or the carbon source used could be explained by the fact that hydrophilic substrates, such as molasses or glycerol, are degraded until forming intermediates of the glycolytic pathway, such as glucose 6-phosphate, which is one of the main precursors of carbohydrates found in the hydrophilic moiety of biosurfactants [64].

Regarding lipid content, glycerol was the substrate for which biosurfactants produced by all the three strains have presented the highest amount of lipids opposite to sugar cane molasses. This difference in biosurfactants composition could be due to the fact that when glycerol is used as substrate, the microbial mechanism could be mainly directed to the lipolytic pathway and gluconeogenesis, thereby allowing its use for the production of fatty acids and sugars [64]. Also, compared to molasses, glycerol is easily oxidised to pyruvate through glycolysis and pyruvate is then converted into acetyl-CoA, which produces malonyl-CoA when united with oxaloacetate, followed by conversion into a fatty acid, which is one of the precursors for the synthesis of lipids [65].

The results of this study show that biosurfactants produced by* Lb. cellobiosus* TM1 and* Lb. delbrueckii *N2 while using sugar cane molasses as substrate could be considered as glycoproteins due to its major content of proteins and sugars. A glycoprotein nature of biosurfactants was also reported elsewhere by Gołek et al. [66] in their study with* L. casei. *In the same way, Tahmourespour et al. [67] and Madhu and Prapulla [24] have, respectively, shown that* Lb. acidophilus *and* Lb. plantarum* CFR2194 produced glycoprotein biosurfactants.

With* Lb. plantarum* G88, the biosurfactants produced while using sugar cane molasses as substrate were composed of proteins (8.96 ± 0.53 g/100 gMS), sugars (51.13 ± 0.92 g/100 gMS), and lipids (39.60 ± 0.65 g/100 gMS), meaning that there are glycolipoprotein nature. This could be due to the wide variability of biosurfactants metabolism with strain and carbon source used [68]. Vecino et al. [25] reported similar observation (glycolipoprotein nature) with biosurfactants produced by* Lb. pentosus.*

Biosurfactants produced with all the three strains while using glycerol as substrate are mainly composed of sugars and lipids. According to Syldatk and Wagner [68], the biosynthesis of a surfactant occurs through different routes which are both dependent on the substrate. A glycolipid nature of lactic acid bacteria biosurfactants was reported in the literature. Partovi et al. [69] reported that* Lactococcus lactis *produced glycolipid biosurfactants. Sauvageau et al. [70] also showed that* Lb. plantarum* IRL 560 produced biosurfactants of glycolipid nature.

#### 3.5.2. Presence of Phosphates in Crude Biosurfactants

In order to complete the nature of biosurfactants produced by the three lactobacilli strains, the presence of phosphates in their composition was sought. Only biosurfactants produced by* Lb. cellobiosus* TM1 and* Lb. delbrueckii *N2 while using glycerol as substrate were positive. This result means that the biosurfactants produced by these strains could be a mixture of compounds containing proteins, sugars, lipids, and phosphates. Other studies in the literature have reported the complex nature of biosurfactants produced by lactic acid bacteria. Velraeds et al. [20] and Rodrigues et al. [32] showed that biosurfactants derived from various lactic acid bacteria are multicomponent compounds, containing proteins, polysaccharides, and phosphate groups. In the same way, biosurfactants derived from lactic acid bacteria strains were described as multicomponent mixtures containing protein fractions, various polysaccharides, and phosphate groups [24, 70].

#### 3.5.3. Emulsifying Activity and Storage Stability of Emulsions

Emulsifying activity is one of the properties that biopreservatives used in food industries must have. This property permits their homogenous distribution in all food matrix and optimizes their efficacity. Emulsifying activity of the crude biosurfactants produced by three strains of lactobacilli with molasses or glycerol as substrate was determined.  Table 9 presents the emulsification index obtained after 1, 24, 48, 72, and 96 hours of storage at room temperature. After 1 hour, an emulsification index ranging from 64.00 ± 2.82% to 91.50 ± 3.70% was observed. The emulsifying activity observed could be due to the fact that adsorption of biosurfactants at the interface between water and oil allows a decrease in energy required to generate interfacial area, thus facilitating obtainment of drops with small diameter during emulsification step and allowing the formation of emulsions.

Except the biosurfactants of* Lb. plantarum* G88 with molasse as substrate, a nonsignificant variation (*p* > 0.05) of emulsification index after 72 h of storage was observed. Therefore, it can be concluded that the crude biosurfactants produced by* Lactobacillus cellobiosus* TM1,* Lactobacillus delbrueckii *N2, and* Lactobacillus plantarum* G88 are able to stabilize emulsions for 72 h. The ability of biosurfactants to stabilize emulsions could be explained by the fact that adsorption of biosurfactants to oil/water interface generated by mechanical energy provided by agitation avoids coalescence of drops by electrostatic repulsion or by establishment of a steric barrier [71]. This permits reducing Ostwald repining and coalescence which are responsible for an irreversible increase of drops size leading to emulsions breakage. The results observed in this study are similar to those reported by Lu [72] who showed that rhamnolipids biosurfactants are able to stabilize oil in water emulsions for 72 hours.

In accordance with Rulison and Lochhead [73] who reported that a good emulsifier must be not only able to slip in between water/oil interfaces but also be anchored in irreversible manner in order to avoid all desorption of polymer at the time which droplets draw nearer one to others, the biosurfactants produced in this study can be considered as good emulsifiers.

However, the emulsifying activity of crude biosurfactants obtained in this study was quite stable after 72 h in comparison to synthetic surfactants SDS where a significant loss of emulsifying activity was noticed after 48 h. This means that the crude biosurfactants produced by lactobacilli strains while using sugar cane molasses or glycerol as substrate can effectively be used to substitute chemical emulsifiers in food industry.

#### 3.5.4. Critical Micelle Concentration

**Figure 3:**
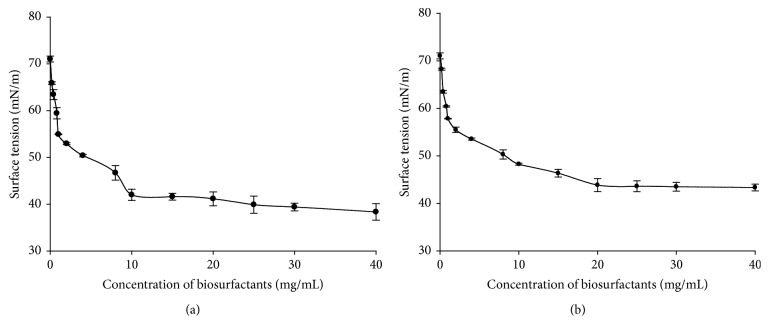
Surface tension values (mN/m) versus biosurfactants concentration (mg/mL) obtained with the biosurfactants produced by* Lb. cellobiosus* TM1 with sugar cane molasses (a) or glycerol (b) as substrate.

**Figure 4:**
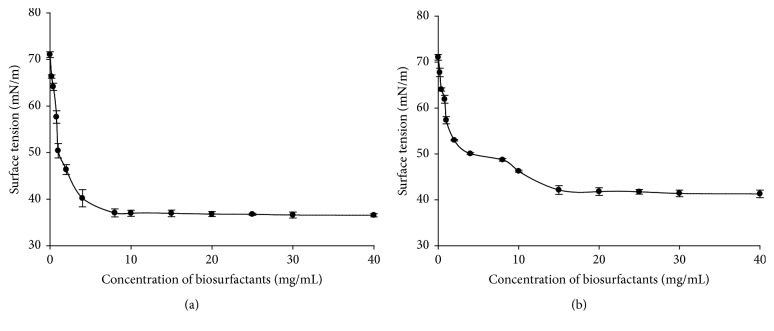
Surface tension values (mN/m) versus biosurfactants concentration (mg/mL) obtained with the biosurfactants produced by* Lb. delbrueckii* N2 with sugar cane molasses (a) or glycerol (b) as substrate.

**Figure 5:**
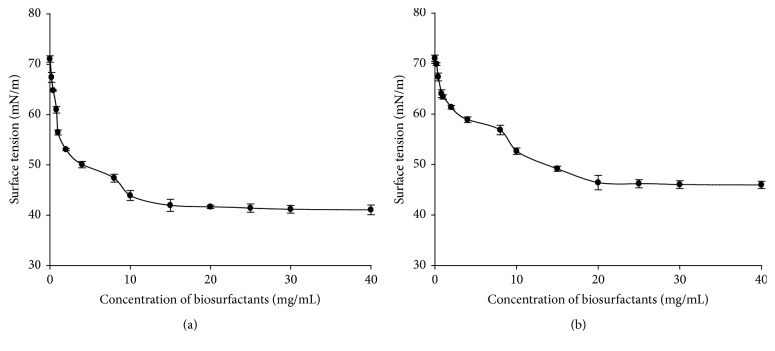
Surface tension values (mN/m) versus biosurfactants concentration (mg/mL) obtained with the biosurfactants produced by* Lb. plantarum* G88 with sugar cane molasses (a) or glycerol (b) as substrate.

It is well known that important interfacial properties (such as detergency and solubilisation) are affected by the existence of micelles in solution [74]. Therefore, the CMC is widely used as index to evaluate the surface activity of a given surfactant. Figures 3, 4, and 5 present the evolution of surface tension versus concentration of biosurfactants produced by* Lactobacillus cellobiosus* TM1,* Lactobacillus delbrueckii* N2, and* Lactobacillus plantarum* G88 while using sugar cane molasses or glycerol as substrate, respectively.

It can be observed from Figures 3(a) and 3(b) that surface tension decreases progressively when the concentration of crude biosurfactants increases. At biosurfactants concentration higher than 10 and 20 mg/mL (resp., on Figures 3(a) and 3(b)) the surface tension becomes stable, and there is no further significant reduction (*p* < 0.05) even at the highest concentrations tested. This means that 10 and 20 mg/mL are, respectively, the CMC of biosurfactants produced by* Lb. cellobiosus* TM1 with sugar cane molasses or glycerol as substrate. Similar phenomena were observed at 8 and 15 mg/mL for* Lb. delbrueckii *N2 with sugar cane molasses or glycerol as substrate, respectively (Figures 4(a) and 4(b)), and at 15 and 20 mg/mL for* Lactobacillus plantarum* G88 with sugar cane molasses or glycerol as substrate, respectively (Figures 5(a) and 5(b)).

The CMC and the surface tension values herein obtained for the three lactobacilli strains are in good agreement with the values reported in the literature for biosurfactants produced by other lactobacilli. Rodrigues et al. [32] found with* Streptococcus thermophilus* A a surface tension of 36.0 mN/m and a CMC of 20.0 mg/mL. Brzozowski et al. [47] obtained with* Lactobacillus fermenti* 126 a surface tension of 45.1 mN/m and a CMC of 9.0 mg/mL. Gudiña et al. [5] reported with* Lactobacillus agilis* CCUG31450 a surface tension of 42.5 mN/m and a CMC of 7.5 mg/mL.

From a practical point of view, it is important to differentiate effective and efficient surfactant. Effectiveness is measured by the minimum value to which the surface tension can be reduced, whereas efficiency is measured by the surfactant concentration required to produce a significant reduction in the surface tension of water, namely, the CMC [75]. The crude biosurfactants of* Lb. delbrueckii *N2 present the lowest CMC value and surface tension reduction independent of substrate used, suggesting that it is more effective and efficient than the biosurfactants produced by the other strains.

## 4. Conclusion

The results of this study have shown the good ability of sugar cane molasse or glycerol to be used as low-cost substrates in the production of biosurfactants by* Lactobacillus delbrueckii* N2,* Lactobacillus cellobiosus* TM1, and* Lactobacillus plantarum* G88. Statistical analysis has shown that the properties of the produced biosurfactants are correlated with the biosurfactants producing strains and the substrate used. A preliminary characterization of the crude biosurfactants obtained has shown that the use of glycerol as substrate mainly stimulates the production of glycolipids biosurfactants, while with sugar cane molasses as substrate, the production of glycoprotein biosurfactants is stimulated. Emulsions obtained with crude biosurfactants are stables after 72 h of storage at room temperature, suggesting that they are effective in forming and stabilizing emulsions. This study suggested the use of sugar cane molasses or glycerol as substrate by lactobacilli strains to produce biosurfactants which can be used in food industry as emulsifier or biopreservatives.

## Figures and Tables

**Table 1:** Physicochemical composition of clarified sugar cane molasses.

Parameters	Values
pH	5.60 ± 0.26
Total dissolved substances (%) or Brix (°)	79.24 ± 0.12
Water content (%)	37.47 ± 0.18
Dry matter (%)	62.52 ± 0.19
Ash content (g/100 gDM)	3.56 ± 0.28
Reducing sugar content (g/100 gDM)	15.36 ± 1.16
Total sugars content (g/100 gDM)	51.23 ± 2.42
Total proteins content (g/100 gDM)	2.59 ± 0.07

^*∗*^DM = dry mater.

**Table 2:** Emulsification index of supernatants obtained with sugar cane molasses or glycerol as substrates.

Substrates	Emulsification index (%)					
	Biosurfactants producing strains			Control		
	*Lb. plantarum* G88	*Lb. delbrueckii* N2	*Lb. cellobiosus* TM1	*Culture medium*	*H*_*2*_ *O*	*SDS 1%* *(w/v)*
Molasses	49.89 ± 5.28^cB^	81.00 ± 1.14^eB^	63.50 ± 4.94^dB^	21.85 ± 1.75^bA^	11.50 ± 2.12^a^	92.25 ± 1.21^f^
Glycerol	41.81 ± 2.56^cA^	61.81 ± 2.56^eA^	53.28 ± 7.91^dA^	19.31 ± 2.40^bA^	11.50 ± 2.12^a^	92.25 ± 1.21^f^

Values are means ± standard deviation; *n* = 3; a, b, c, and so on indicated column comparison; A, B, C, and so on indicated line comparison; values followed by the same lowercase letter in superscript on the same line or by the same capital letter in superscript on the same column are not significantly different (*p* < 0.05) according to Duncan's multiple range test. The emulsion has been obtained by mixing supernatant of culture with refined palm oil.

**Table 3:** Drop diameters of supernatants obtained with sugar cane molasses or glycerol as substrates.

Substrates	Drop diameters (mm)					
	Biosurfactants producing strains			Control		
	*Lb. plantarum* G88	*Lb. delbrueckii* N2	*Lb. cellobiosus* TM1	*Culture medium*	*H*_*2*_ *O*	*SDS 1%* *(w/v)*
Molasses	17.50 ± 0.70^dA^	19.00 ± 1.41^dA^	14.50 ± 0.70^cB^	12.33 ± 1.50^b^	9.50 ± 1.41^a^	22.50 ± 0.70^e^
Glycerol	16.20 ± 0.84^dA^	18.55 ± 0.63^eA^	12.80 ± 0.98^cA^	9.92 ± 0.50^b^	9.50 ± 1.41^a^	22.50 ± 0.70^f^

Values are means ± standard deviation; *n* = 3; a, b, c, and so on indicated column comparison; A, B, C, and so on indicated line comparison; values followed by the same lowercase letter in superscript on the same line or by the same capital letter in superscript on the same column are not significantly different (*p* < 0.05) according to Duncan's multiple range test.

**Table 4:** Surface tension of supernatants obtained with sugar cane molasses or glycerol as substrates.

Substrates	Surface tension (mN/m)					
	Biosurfactants producing strains			Control		
	*Lb. plantarum* G88	*Lb. delbrueckii* N2	*Lb. cellobiosus* TM1	*Culture medium*	*H*_*2*_ *O*	*SDS 1%* *(w/v)*
Molasses	47.50 ± 1.78^dA^	41.90 ± 0.79^bA^	44.20 ± 0.37^cA^	59.71 ± 0.50^eA^	73.00 ± 0.77^f^	31.98 ± 2.68^a^
Glycerol	49.00 ± 2.43^cA^	46.20 ± 1.64^bB^	48.50 ± 1.33^bB^	60.17 ± 0.14^dB^	73.00 ± 0.77^e^	31.89 ± 2.68^a^

Values are means ± standard deviation; *n* = 3; a, b, c, and so on indicated column comparison; A, B, C, and so on indicated line comparison; values followed by the same lowercase letter in superscript on the same line or by the same capital letter in superscript on the same column are not significantly different (*p* < 0.05) according to Duncan's multiple range test.

**Table 5:** Inhibition diameters of supernatants obtained with sugar cane molasses or glycerol as substrates.

Tests germs	Inhibition diameters (mm)					
	Molasses			Glycerol		
	*Lb. delbrueckii* N2	*Lb. cellobiosus* TM1	*Lb. plantarum* G88	*Lb. delbrueckii* N2	*Lb. cellobiosus* TM1	*Lb. plantarum* G88
*Bacillus* sp. BC1	27.00 ± 4.24^deA^	24.50 ± 6.36^cdeA^	23.00 ± 9.89^cdA^	57.50 ± 3.53^gC^	46.50 ± 2.12^gB^	41.00 ± 1.41^fA^
*Escherichia coli* E2B	17.00 ± 1.41^bA^	27.50 ± 3.53^cdeC^	22.00 ± 1.41^cB^	48.50 ± 2.12^fC^	33.50 ± 2.13^fB^	19.00 ± 1.50^bA^
*Escherichia coli* E2R	29.00 ± 1.41^eA^	26.50 ± 2.12^deA^	27.50 ± 3.53^dA^	18.50 ± 2.30^abA^	16.00 ± 1.50^bcA^	31.00 ± 4.24^deB^
*Escherichia coli* E5	18.00 ± 2.82^bcA^	27.00 ± 2.82^eB^	17.00 ± 2.82^abA^	21.50 ± 2.50^bcC^	12.50 ± 0.70^aA^	15.00 ± 1.40^aB^
*Escherichia coli* E6	20.50 ± 0.70^cA^	21.00 ± 1.41^bA^	32.00 ± 2.82^dB^	22.00 ± 2.82^bcA^	32.50 ± 2.30^efB^	34.00 ± 2.00^eB^
*Candida albicans* LV1	12.50 ± 0.70^aA^	13.00 ± 2.82^aA^	16.00 ± 2.82^abA^	16.50 ± 0.70^aB^	12.00 ± 1.10^aA^	11.50 ± 2.34^aA^
*Pseudomonas putida* PS3	17.00 ± 2.83^bcA^	17.00 ± 2.80^abA^	23.50 ± 2.12^cB^	28.50 ± 2.13^dB^	14.00 ± 1.30^aA^	15.50 ± 2.12^aA^
*Pseudomonas aeruginosa* PSB1	23.00 ± 2.90^cdB^	20.50 ± 0.70^bAB^	18.50 ± 0.70^bA^	23.50 ± 2.31^cB^	14.50 ± 2.12^abA^	14.50 ± 0.70^aA^
*Pseudomonas aeruginosa* PSB2	14.00 ± 1.45^abA^	16.00 ± 2.85^abAB^	19.50 ± 0.80^bB^	18.50 ± 2.40^abA^	26.00 ± 1.50^dB^	26.00 ± 2.90^cB^
*Pseudomonas putida* PSJ1	18.50 ± 2.12^bcA^	19.50 ± 2.12^bA^	32.00 ± 2.85^dB^	26.50 ± 0.70^cdB^	24.00 ± 1.42^dB^	20.50 ± 0.71^bA^
*Pseudomonas aeruginosa* PSR1	22.00 ± 2.87^cdA^	23.50 ± 0.70^cdA^	21.50 ± 4.94^bcA^	18.50 ± 2.17^aA^	32.50 ± 3.53^efC^	24.50 ± 8.80^abcdB^
*Pseudomonas aeruginosa* PSR2	19.50 ± 2.18^bcA^	23.50 ± 2.12^cdA^	19.50 ± 2.12^bcA^	21.00 ± 1.50^bcA^	31.00 ± 1.41^eB^	31.00 ± 1.45^dB^
*Pseudomonas putida* PSV1	25.50 ± 3.53^dA^	27.50 ± 3.53^deA^	32.00 ± 2.82^dB^	20.50 ± 0.70^bA^	28.00 ± 2.82^deB^	22.50 ± 2.14^cA^
*Salmonella* sp. S1R	36.00 ± 1.42^fB^	16.50 ± 2.12^abA^	14.00 ± 4.24^abA^	18.50 ± 2.12^abAB^	18.90 ± 1.55^cB^	12.50 ± 4.24^aA^
*Salmonella* sp. S5	37.50 ± 3.56^fB^	14.50 ± 2.20^aA^	14.50 ± 2.12^aA^	41.00 ± 1.41^eC^	26.50 ± 2.12^dB^	14.50 ± 0.76^aA^
*Salmonella* sp. SL2	17.50 ± 3.50^bcA^	21.00 ± 1.41^bcA^	41.00 ± 1.41^eB^	25.50 ± 2.12^cdB^	19.00 ± 1.41^cA^	23.50 ± 2.12^cB^
*Staphylococcus aureus* STP1	23.50 ± 9.94^bcdeA^	28.50 ± 2.12^eA^	31.00 ± 1.41^dA^	56.50 ± 2.12^gC^	36.00 ± 1.41^fA^	36.50 ± 2.20^eA^

Values are means ± standard deviation; *n* = 3; a, b, c, and so on indicated column comparison; A, B, C, and so on indicated line comparison; values followed by the same lowercase letter in superscript on the same column or by the same capital letter in superscript on the same line are not significantly different (*p* < 0.05) according to Duncan's multiple range test.

**Table 6:** Yields of crude biosurfactants obtained with the different substrates.

Biosurfactants producing strains	Yield (g/L)		
	Molasses	Glycerol	MRS broth
*Lb. plantarum* G88	2.43 ± 0.09^aB^	2.32 ± 0.19^aB^	0.30 ± 0.02^aA^
*Lb. delbrueckii* N2	3.03 ± 0.09^cC^	2.77 ± 0.03^bB^	0.51 ± 0.09^bA^
*Lb. cellobiosus* TM1	2.79 ± 0.06^bB^	2.82 ± 0.05^cC^	0.49 ± 0.07^bA^

These organic extracts are crude biosurfactants; Values are means ± standard deviation; *n* = 3; a, b, c, and so on indicated column comparison; A, B, C, and so on indicated line comparison; values followed by the same lowercase letter in superscript on the same line or by the same capital letter in superscript on the same column are not significantly different (*p* < 0.05) according to Duncan's multiple range test.

**Table 7:** Pearson correlation matrix of the different variables.

Variables	E24	Surface tension	Drop diameters	Inhibition diameters	Yield
E24	1				
Surface tension	−0.953^*∗*^	1			
Drop diameters	0.955^*∗*^	−0.877^*∗*^	1		
Inhibition diameters	−0.202	0.445	−0.135	1	
Yield	0.895^*∗*^	−0.754	0.839^*∗*^	0.225	1

*∗* indicates significant correlation (*p* < 0.05).

**Table 8:** Chemical composition of biosurfactants produced by lactobacilli strains while using sugar cane molasses or glycerol as substrate.

Substrates	LAB strains	Total proteins (g/100 gMS)	Total sugars (g/100 gMS)	Total lipids (g/100 gMS)
Molasses	*Lb. cellobiosus* TM1	52.93 ± 1.27^e^	46.66 ± 0.47^e^	0.00 ± 0.00
	*Lb. delbrueckii *N2	63.64 ± 0.24^f^	35.26 ± 1.10^c^	1.10 ± 0.70^a^
	*Lb. plantarum* G88	8.96 ± 0.53^d^	51.13 ± 0.92^f^	39.60 ± 0.65^b^

Glycerol	*Lb. cellobiosus* TM1	4.20 ± 0.51^c^	27.10 ± 0.36^a^	68.20 ± 1.96^d^
	*Lb. delbrueckii *N2	3.18 ± 0.94^b^	31.26 ± 1.36^b^	65.23 ± 1.07^d^
	*Lb. plantarum* G88	1.20 ± 0.14^a^	43.96 ± 1.40^d^	54.40 ± 0.30^d^

LAB = lactic acid bacteria; values are means ± standard deviation; *n* = 3; a, b, c, and so on indicated column comparison; values followed by the same letter in superscript on the same column are not significantly different (*p* < 0.05) according to Duncan's multiple range test.

**Table 9:** Emulsifying activity of biosurfactants produced by the three *Lactobacillus* strains while using sugar cane molasse and glycerol as substrate.

Substrates	LAB strains	Emulsification index (%)				
		Time (hours)				
		1 h	24 h	48 h	72 h	96 h
Molasses	*Lb. *TM1	89.00 ± 4.24^b^	88.50 ± 2.92^b^	86.5 ± 2.12^b^	83.00 ± 2.41^b^	64.50 ± 0.70^a^
	*Lb*. N2	78.00 ± 3.50^b^	77.25 ± 1.06^b^	75.50 ± 0.70^b^	73.00 ± 2.82^b^	61.00 ± 3.50^a^
	*Lb. *G88	70.20 ± 0.40^d^	68.00 ± 1.41^c^	66.50 ± 1.41^c^	52.50 ± 2.12^b^	46.50 ± 2.12^a^

Glycerol	*Lb*. TM1	73.00 ± 2.82^b^	72.00 ± 1.41^b^	69.00 ± 4.24^b^	67.00 ± 3.41^b^	50.50 ± 2.12^a^
	*Lb*. N2	91.50 ± 3.70^b^	89.00 ± 1.41^b^	87.00 ± 2.42^b^	85.50 ± 3.12^b^	67.50 ± 3.53^a^
	*Lb*. G88	64.00 ± 2.82^b^	63.50 ± 0.70^b^	61.50 ± 3.53^b^	59.50 ± 2.70^b^	49.00 ± 1.41^a^

Control						

SDS 1% (w/v)		91.00 ± 1.41^d^	89.50 ± 0.70^d^	85.00 ± 1.41^c^	65.00 ± 2.82^b^	60.50 ± 0.70^a^

Distilled water		11.50 ± 0.12	0.0 ± 0.0	0.0 ± 0.0	0.0 ± 0.0	0.0 ± 0.0

LAB = lactic acid bacteria; *Lb. *TM1 = *Lactobacillus cellobiosus* TM1; *Lb. *N2 = *Lactobacillus delbrueckii *N2; *Lb. *G88 = *Lactobacillus plantarum* G88; values are means ± standard deviation; *n* = 3; a, b, c, and so on indicated column comparison; values followed by the same letter in superscript on the same line are not significantly different (*p* < 0.05) according to Duncan's multiple range test.
